# Na_v_1.5 regulates breast tumor growth and metastatic dissemination *in vivo*

**DOI:** 10.18632/oncotarget.5441

**Published:** 2015-10-06

**Authors:** Michaela Nelson, Ming Yang, Rebecca Millican-Slater, William J. Brackenbury

**Affiliations:** ^1^ Department of Biology, University of York, Heslington, York, YO10 5DD, UK; ^2^ Department of Histopathology, St James's University Hospital, Leeds, LS9 7TF, UK

**Keywords:** adhesion, breast cancer, invasion, metastasis, voltage-gated Na^+^ channel

## Abstract

Voltage-gated Na^+^ channels (VGSCs) mediate action potential firing and regulate adhesion and migration in excitable cells. VGSCs are also expressed in cancer cells. In metastatic breast cancer (BCa) cells, the Na_v_1.5 α subunit potentiates migration and invasion. In addition, the VGSC-inhibiting antiepileptic drug phenytoin inhibits tumor growth and metastasis. However, the functional activity of Na_v_1.5 and its specific contribution to tumor progression *in vivo* has not been delineated. Here, we found that Na_v_1.5 is up-regulated at the protein level in BCa compared with matched normal breast tissue. Na^+^ current, reversibly blocked by tetrodotoxin, was retained in cancer cells in tumor tissue slices, thus directly confirming functional VGSC activity *in vivo*. Stable down-regulation of Na_v_1.5 expression significantly reduced tumor growth, local invasion into surrounding tissue, and metastasis to liver, lungs and spleen in an orthotopic BCa model. Na_v_1.5 down-regulation had no effect on cell proliferation or angiogenesis within the in tumors, but increased apoptosis. *In vitro*, Na_v_1.5 down-regulation altered cell morphology and reduced CD44 expression, suggesting that VGSC activity may regulate cellular invasion *via* the CD44-src-cortactin signaling axis. We conclude that Na_v_1.5 is functionally active in cancer cells in breast tumors, enhancing growth and metastatic dissemination. These findings support the notion that compounds targeting Na_v_1.5 may be useful for reducing metastasis.

## INTRODUCTION

Metastasis is the main cause of morbidity and mortality from solid tumors, including breast cancers (BCa) [1, 2]. Thus, there is a an urgent need to better understand the mechanism(s) involved in metastasis in order to identify, characterize and validate new molecular targets [3].

Various classes of ion channels, including voltage-gated Na^+^ channels (VGSCs), play major roles in cancer progression [4, 5]. VGSCs are heteromeric protein complexes containing pore-forming α subunits (Na_v_1.1-Na_v_1.9) and smaller, non-pore-forming β subunits (β1-β4), which are also cell adhesion molecules (CAMs) [6]. VGSCs are classically responsible for the influx of Na^+^ underlying the action potential in electrically excitable cells. VGSCs are therefore well-established clinical targets for the treatment of a range of neurological disorders [7]. VGSC α and β subunits play a critical role during central nervous system (CNS) development, regulating electrical excitability, proliferation, fasciculation, neurite outgrowth, pathfinding and migration [8]. In addition, the Na_v_1.5 α subunit (gene, *SCN5A*) is highly expressed in the heart, where it underlies the cardiac action potential, is required for normal heart development, and is the target of a number of antiarrhythmic drugs [9–11]. Thus, regulation of tissue and organ development may be a general feature of VGSCs, both in the CNS and the heart. Emerging evidence suggests that VGSCs are also widely expressed in cells traditionally considered to be non-excitable, including astrocytes, fibroblasts, immune cells, microglia and cancer cells [12].

Na_v_1.5 is expressed in metastatic triple-negative (lacking estrogen receptor, progesterone receptor, and HER2) MDA-MB-231 cells, where it enhances migration and invasion through an extracellular matrix *in vitro* [13, 14]. Similar findings in cells from other cancer types suggest that VGSC activity may be a general feature of tumors [4, 15]. *SCN5A* mRNA is up-regulated in breast tumors compared to normal breast tissue, and associates with recurrence, metastasis and reduced survival [14, 16]. Interestingly, in BCa cells, Na_v_1.5 is predominantly expressed in its neonatal D1:S3 splice form, and it is this splice variant that is responsible for VGSC-dependent invasion [14, 17]. Na^+^ current carried by Na_v_1.5 potentiates invasion *via* regulation of the Na^+^/H^+^ exchanger, NHE1, resulting in local extracellular acidification and activation of pH-dependent cysteine cathepsins [18–20]. In addition, Na_v_1.5 is a key regulator of an invasion-promoting gene network in colorectal cancer cells [21]. The VGSC β1 subunit is also up-regulated in BCa, and increases tumor growth and metastasis [22]. Thus, VGSC α and β subunits may both play a role in cancer progression. We have found that the VGSC-inhibiting Class Ib antiarrhythmic agent and antiepileptic drug phenytoin significantly reduces Na^+^ current in MDA-MB-231 cells *in vitro* [16], and reduces proliferation, tumor growth and metastasis *in vivo* [23]. However, the specific contributions of Na_v_1.5 to tumor growth, invasion and metastasis *in vivo* have not been previously investigated.

The purpose of the present study was to investigate the specific involvement of Na_v_1.5 in BCa progression *in vivo*. We show that Na_v_1.5 is up-regulated at the protein level in human BCa samples compared with normal breast tissue. In addition, using slice recording, we show for the first time that Na^+^ currents exist in tumor tissue, confirming that VGSCs are functionally active in acutely prepared *ex vivo* tumor tissue preparations. Furthermore, stable down-regulation of Na_v_1.5 using lentiviral shRNA significantly reduces tumor growth, local invasion and metastasis *in vivo*. We propose that Na_v_1.5 is functionally active in breast tumors, enhancing both tumor growth and metastasis. These findings suggest that Na_v_1.5 should be further studied both as a potential biomarker and a therapeutic target.

## RESULTS

### Na_v_1.5 is up-regulated in breast tumors

**Figure 1:**
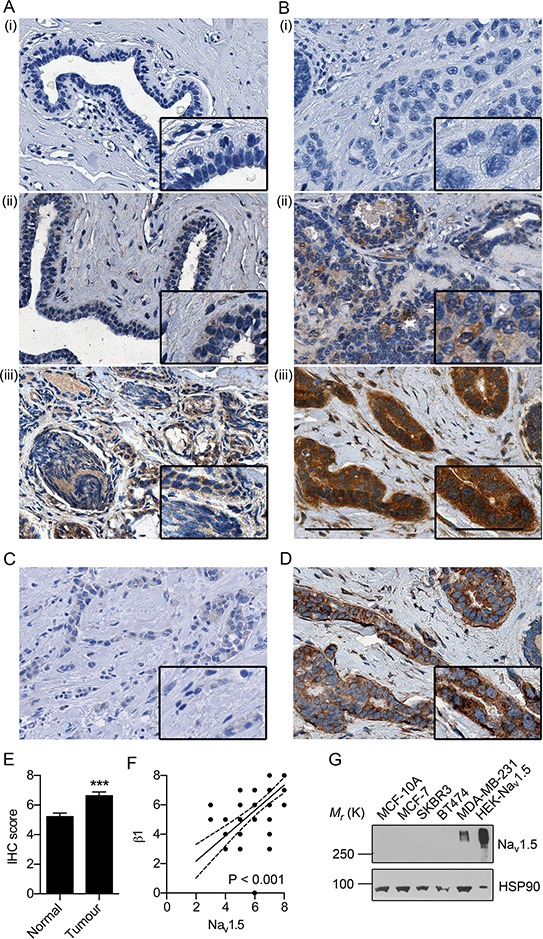
Na_v_1.5 expression in breast cancer **A.** Representative images of non-cancer breast tissue and **B.** breast tumor tissue in which Na_v_1.5 was (i) absent, (ii) weakly positive, and (iii) strongly positive. **C.** Absence of immunoreactivity in a “strongly positive” tumor stained with anti-Na_v_1.5 antibody preincubated with immunizing peptide. **D.** Adjacent section from the same tumor in (Biii) stained with anti-β1 antibody [22]. Scale bar, 100 μm. Insets, higher magnification images of tumor cells, scale bar, 50 μm. **E.** Mean Allred score for matched normal breast and tumor samples. Data are mean + SEM (*n* = 36 matched cases). ***P < 0.001. **F.** Na_v_1.5 vs. β1 IHC score [22] in adjacent sections from matched tumor samples. Pearson *r* = 0.66 (*P* < 0.001). Solid line, linear regression; broken lines 95% confidence intervals. **G.** Western blot of Na_v_1.5 expression across a panel of BCa cell lines. Loading control = heat shock protein 90 (HSP90). Positive control = HEK293 cells stably expressing Na_v_1.5 [56].

We have previously shown that *SCN5A* is up-regulated at the mRNA level in breast tumors compared to normal, non-cancer tissue [16]. A small qualitative study (*n* = 10) revealed a similar up-regulation of expression of the neonatal Na_v_1.5 splice variant at the protein level [14]. Here, we studied the expression of Na_v_1.5 at the protein level in human tissue samples by immunohistochemistry (IHC), using an antibody that recognizes both adult and neonatal splice variants [21]. Na_v_1.5 was expressed in the cytoplasm and at the plasma membrane of normal epithelial and carcinoma cells (Figure 1A, 1B). Antibody specificity was confirmed in breast tumor tissue and rat heart tissue, where Na_v_1.5 is highly expressed, by absence of staining following pre-incubation with the immunizing peptide (Figure 1C and Supplementary Figure S1A–S1C). Importantly, Na_v_1.5 expression was significantly higher in tumor than in matched surrounding non-cancer breast tissue (*P* < 0.001; Figure 1E). Interestingly, the proportion of cases with a recorded lymph node metastasis was ~3-fold larger for tumors with high Na_v_1.5 expression, than for those with low Na_v_1.5 expression, although this was not statistically significant (*P* = 0.19; Supplementary Table S1). The Na_v_1.5 expression level in the primary tumor did not correlate with age, ER status, grade, menopausal status, or 5-year BCa-specific survival (Supplementary Table S1). However, the Na_v_1.5 expression level strongly correlated with β1 expression in adjacent sections from the same tumor samples (*P* < 0.001; Figure 1Biii, 1D, 1F) [22]. Western blotting across a panel of BCa cell lines and the non-cancer mammary epithelial cell line MCF-10A revealed that Na_v_1.5 is highly expressed in the strongly metastatic MDA-MB-231 cell line, but is not detected in other, less invasive BCa or normal epithelial cell lines (Figure 1G). This is consistent with previous observations indicating that the neonatal splice variant of Na_v_1.5 is absent from MCF-7 cells, but is present in MDA-MB-231 cells [14]. Interestingly, in contrast to the tumor specimens, Na_v_1.5 expression in these cell lines does not match that of β1, which we showed previously to be most highly expressed in MCF-7 cells [22, 24]. Together, these data suggest that Na_v_1.5 is up-regulated in a subset of breast tumors at the protein level and its expression may associate with β1 in some tumors.

### Na^+^ current is retained in tumors *in vivo*

**Figure 2:**
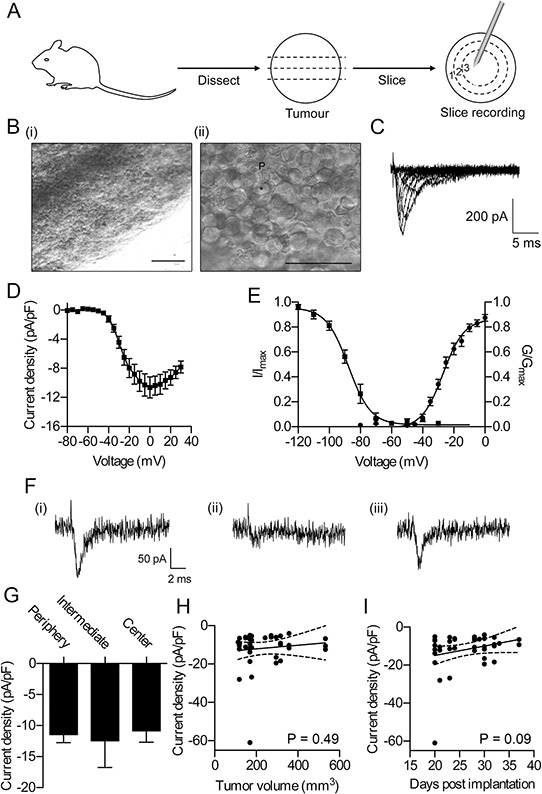
Functional Na^+^ currents are retained in tumors *in vivo* **A.** Tumor preparation for slice recording. Recordings were taken from cells in (1) the periphery (≤1 mm from tumor surface, (2) the intermediate zone (>1 mm and ≤ 1.5 mm from surface), and (3) the center (>1.5 mm from surface). **B.** Representative (i) low magnification and (ii) high magnification bright field images of upper surface of tumor slice prepared for recording. P, location of patch pipette, *, location of recorded cell. Scale bars, 100 μm in (i) and 50 μm in (ii). **C.** Typical whole-cell recording from tumor cell in a tissue slice after depolarization to voltages in the range −60 to +30 mV in 10 mV steps following a 250 ms prepulse at −120 mV. **D.** Current-voltage relationship of Na^+^ currents recorded from cells in tumor slices (*n* = 39, pooled across all regions). **E.** Activation and steady-state inactivation of Na^+^ currents recorded from cells in tumor slices. Normalized conductance (G/G_max_) was calculated from the current data and plotted as a function of voltage. Normalized current (I/I_max_), elicited by test pulses at −10 mV following 250 ms conditioning voltage pulses between −120 and −10 mV, was plotted as a function of the prepulse voltage. Data are fitted with Boltzmann functions (*n* ≥ 14). **F.** Typical whole-cell recordings from tumor cell in a tissue slice following depolarization to −10 mV in control solution (i), following perfusion with 30 μM tetrodotoxin (TTX; ii), and washout (iii). **G.** Peak Na^+^ current density recorded from cells in the three indicated tumor regions (*n* = 20 in periphery, *n* = 12 in intermediate region, *n* = 7 in center). **H.** Peak Na^+^ current density of cells in tumor slices plotted *vs*. tumor volume. Pearson *r* = 0.11 (*P* = 0.49). **I.** Peak Na^+^ current density of cells in tumor slices plotted *vs*. number of days following implantation of tumor cells that recordings were made. Pearson *r* = 0.27 (*P* = 0.09). For (D) (E) and (G), data are mean ± SEM. For (H) and (I), solid line, linear regression; broken lines 95% confidence intervals.

Na^+^ current carried by VGSCs has been detected by whole-cell patch clamp recording of cultured cell lines from a number of different cancers [13, 14, 21, 25–29], providing direct evidence for functional VGSC expression in metastatic cancer cells *in vitro*. In addition, treatment of prostate tumor-bearing rats with tetrodotoxin (TTX) [30], and breast tumor-bearing mice with phenytoin [23] or ranolazine [31] reduces metastasis, providing indirect evidence for VGSC function in metastatic tumors *in vivo*. However, to date, no group has directly studied functional VGSC activity in tumor tissue. To address this gap, we next used whole-cell patch clamping to record membrane Na^+^ currents in tissue slices prepared from tumors 20–37 days after orthotopic implantation of MDA-MB-231 breast cancer cells (Figure 2A). We recorded from cells located on the upper surface of tissue slices at various distances from the edge of the tumor (Figure 2B). Tumor cells displayed fast inward Na^+^ currents *ex vivo* that were similar to currents detected in MDA-MB-231 cells *in vitro* (39/60 cells recorded; Figure 2C–2E) [13, 14, 16, 17]. Importantly, TTX (30 μM) reversibly inhibited the Na^+^ currents, thus confirming these as VGSC currents (Figure 2F). We next compared the peak Na^+^ current density of cells at the tumor periphery (≤1 mm from the lateral surface of the tissue slice, with cells located deeper within the tumor (>1 mm and ≤1.5 mm from the tumor surface, and >1.5 mm from the surface). We found that there was no significant difference in peak Na^+^ current density between cells across these regions (Figure 2G). Nor was there any difference in membrane voltage, membrane capacitance, or other Na^+^ current parameters (Table 1), suggesting that functional VGSC expression is broadly similar between tumor cells located in different regions of the tumor. Finally, there was no relationship between peak Na^+^ current density and tumor size, or stage at which the recordings were taken following orthotopic implantation of the tumor cells (Figure 2H, 2I). These findings demonstrate that Na^+^ current is retained on MDA-MB-231 cells in orthotopic tumors *in vivo*, and expression is broadly similar in cells in different tumor regions/sizes/stages. We postulated that Na_v_1.5 may be responsible for this Na^+^ current in tumor slices and may potentiate tumor progression *in vivo*, as it does *in vitro*. We next tested these possibilities.

**Table 1:** Na^+^ current characteristics in tumor regions

Parameter	Periphery	Intermediate	Center	*P*
C_m_ (pF)	11.8 ± 0.6	11.4 ± 0.9	12.1 ± 1.1	0.87
V_m_ (mV)	−7.6 ± 0.6	−8.8 ± 1.3	−7.2 ± 0.3	0.53
V_a_ (mV)	−31.0 ± 1.8	−27.9 ± 3.1	−27.9 ± 0.4	0.55
V_p_ (mV)	9.8 ± 1.9	10.0 ± 3.4	9.3 ± 3.2	0.99
Activation V_1/2_ (mV)	−27.3 ± 0.9	−26.9 ± 1.8	−26.2 ± 1.8	0.88
Activation *k* (mV)	7.1 ± 0.8	7.0 ± 1.5	5.7 ± 1.5	0.74
Inactivation V_1/2_ (mV)	−85.6 ± 6.1	−90.0 ± 1.0	−89.1 ± 1.5	0.69
Inactivation *k* (mV)	−13.0 ± 5.1	−4.5 ± 1.0	−5.9 ± 1.3	0.20
T_p_ at 0 mV (ms)	1.41 ± 0.11	1.51 ± 0.41	1.01 ± 0.11	0.56

Abbreviations: V_a_, activation voltage; V_p_ voltage at current peak; V_1/2_, half (in)activation voltage; *k*, slope factor; T_p_ time to peak. Periphery: ≤ 1 mm from tumor surface; intermediate zone: >1 mm and ≤ 1.5 mm from surface; centre: >1.5 mm from surface. Data are mean ± SEM.

### Stable down-regulation of Na_v_1.5 in MDA-MB-231 cells

**Figure 3:**
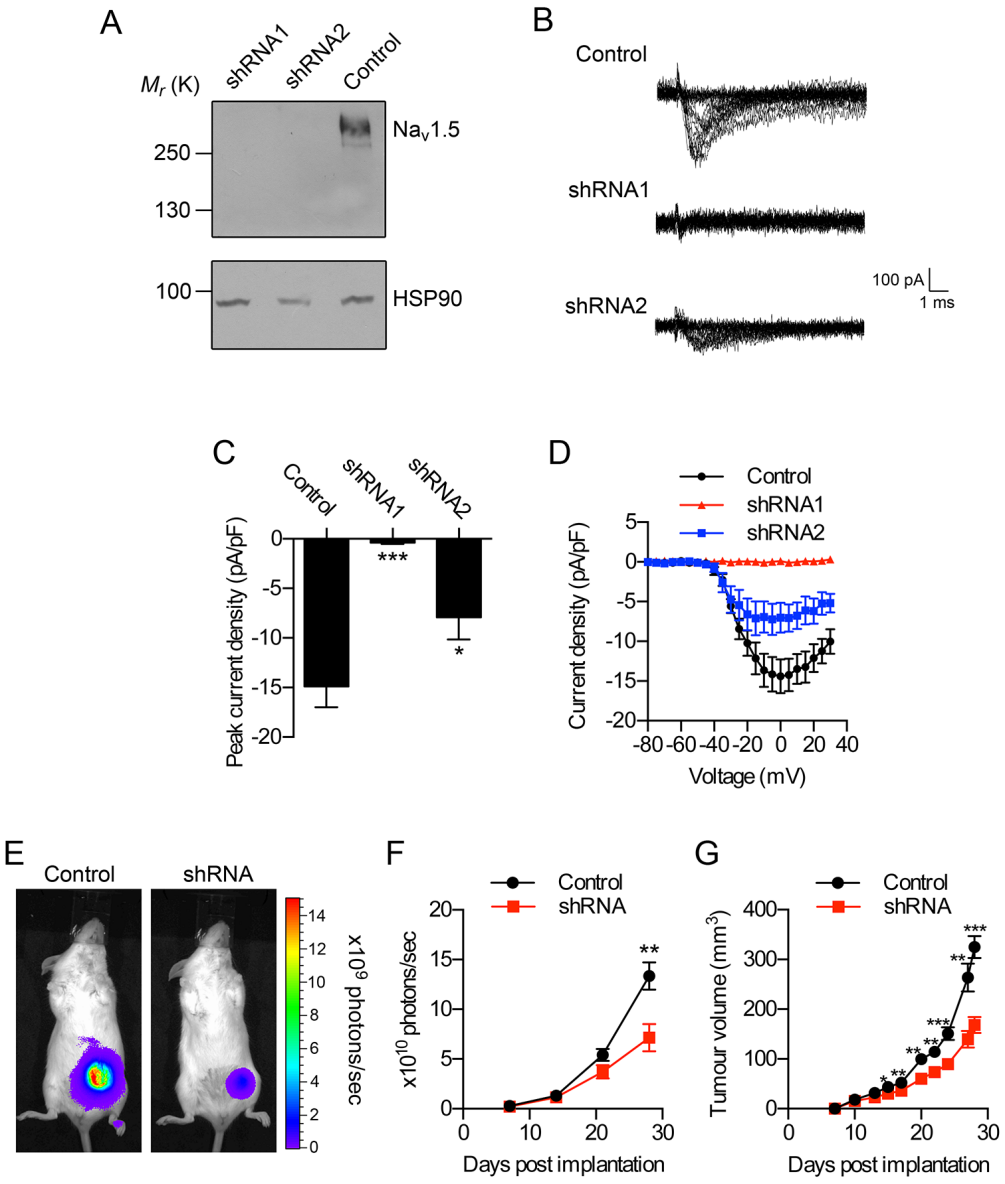
Effect of Na_v_1.5 on breast tumor growth *in vivo* **A.** Western blot of Na_v_1.5 in MDA-MB-231 cells stably expressing control shRNA, or shRNA1, or shRNA2. Loading control = heat shock protein 90 (HSP90). **B.** Typical *in vitro* whole-cell recordings from MDA-MB-231 cells stably expressing control shRNA, or shRNA1, or shRNA2. Recordings were taken after depolarization to voltages in the range −80 to +30 mV in 5 mV steps following a 250 ms prepulse at −120 mV. **C.** Peak Na^+^ current density and **D.** current-voltage relationship of MDA-MB-231 cells stably expressing control shRNA, or shRNA1, or shRNA2 (*n* = 8/each). **E.** Representative bioluminescent images of mice bearing tumors containing MDA-MB-231 cells stably expressing control shRNA (“Control”) and MDA-MB-231 cells expressing shRNA1 (“shRNA”), 4 weeks after implantation. **F.** Bioluminescence measured from primary tumors on the indicated days post-implantation (*n* ≥ 13). **G.** Calculated volume derived from caliper measurement of primary tumors over the same period (*n* ≥ 13). Data are mean ± SEM; **P < 0.01; ***P < 0.001.

In order to study the specific involvement of Na_v_1.5 in tumor progression *in vivo*, we stably down-regulated its expression in MDA-MB-231 cells using lentiviral shRNA. We initially characterized four different shRNAs that each targeted both the neonatal and adult splice variants of Na_v_1.5, compared to a control non-targeting lentiviral shRNA. All four shRNAs reduced the mRNA level of both Na_v_1.5 splice variants (Supplementary Figure S2A). ShRNA1 and shRNA2 were the most effective, and following selection of transduced clones, both shRNAs significantly reduced the mRNA level of both Na_v_1.5 splice variants by ~65–80% (*P* < 0.05; Supplementary Figure S2B). The β1 mRNA level was also lower in shRNA1-transduced cells, although this was not statistically significant (Supplementary Figure S2C). Both shRNA1 and shRNA2 significantly reduced the total cellular Na_v_1.5 protein level to a level sub-detectable by western blot (Figure 3A).

Whole-cell patch clamp recording revealed that both shRNA1 and shRNA2 significantly reduced Na^+^ currents compared to control shRNA-expressing cells (Figure 3B–3D). However, shRNA1 was considerably more effective, reducing peak Na^+^ current density by 97% *vs*. 47% for shRNA2 (*P* < 0.001 and *P* < 0.05, respectively; Figure 3C, 3D). Both shRNA1 and shRNA2 slightly but significantly reduced *in vitro* proliferation by ~10% (*P* < 0.05; Supplementary Figure S3A), although they had no effect on apoptosis measured by *in vitro* TUNEL assay (Supplementary Figure S3B). Both shRNA1 and shRNA2 moderately but significantly reduced cellular migration measured using an *in vitro* wound-healing assay by ~10% (*P* < 0.001 and *P* < 0.01, respectively; Supplementary Figure S4A). Interestingly, shRNA1 significantly reduced *in vitro* invasion by 53% (*P* < 0.001; Supplementary Figure S3B), consistent with previous reports [13, 14, 17, 19], but shRNA2 had no effect (Supplementary Figure S4B). Together, these findings suggest that the relationship between Na_v_1.5 and cellular invasion may be both steep [17] and threshold-dependent, such that a minimum level of channel knock-down/inhibition (e.g. ≥ 60% [14, 17, 18]) is required to elicit an effect on invasion. Alternatively, lentiviral transduction of shRNA2 may have led to the selection of cancer cells in which invasion is less dependent on Na_v_1.5. Given these possibilities, we focused on the more effective shRNA1 for subsequent *in vivo* analyses.

### Na_v_1.5 down-regulation reduces tumor growth and local invasion *in vivo*

We next investigated the specific effect of Na_v_1.5 on tumor growth and invasion *in vivo*. Luciferase-expressing MDA-MB-231 cells stably expressing non-targeting control shRNA (“control cells”) or shRNA1 (“shRNA cells”) were orthotopically implanted into the inguinal mammary fat pads of female *Rag2^−/−^ Il2rg^−/−^* mice and tumor growth was monitored by non-invasive bioluminescent imaging. Importantly, luciferase activity was very similar in both control and shRNA cells (Supplementary Figure S5A, S5B). Photon flux from shRNA tumors increased more slowly than for control tumors, reaching statistical significance after 4 weeks (*P* < 0.01; Figure 3E, 3F). Concurrent monitoring of tumor growth by caliper measurement revealed a similar relationship that became statistically significant after 2 weeks, thus confirming the bioluminescent data (*P* < 0.05; Figure 3G).

**Figure 4:**
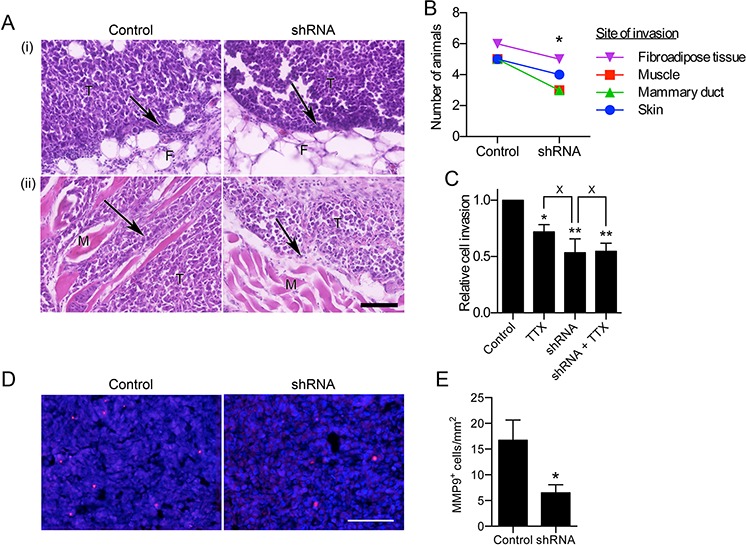
Effect of Na_v_1.5 on local invasion from the primary tumor **A.** Images of tissue sections from tumors containing MDA-MB-231 cells stably expressing control shRNA (“Control”) and MDA-MB-231 cells expressing shRNA1 (“shRNA”), stained with H&E showing (i) mammary fat pad and (ii) skeletal muscle invasion. Arrows, infiltration of tumor cells (T) into fibroadipose tissue (F) or skeletal muscle fibers (M) Scale bar, 100 μm. **B.** Number of animals in the experiment whose tumors displayed invasion at the indicated sites, detected by H&E staining (*n* = 7 for both control and shRNA). **C.** *In vitro* invasion of control and cells ± TTX (30 μM) for 24 h (*n* = 4; **P < 0.01; *P < 0.05; Neuman-Keuls test) **D.** Tumor sections stained with anti-MMP9 (red) and DAPI (blue). **E.** MMP9^+^ cells/mm^2^ (*n* = 7). Data are mean and SEM.

H&E staining revealed that both control and shRNA tumors were broadly similar, and contained some local invasion into surrounding tissue. Importantly, this invasion was noticeably reduced in shRNA tumors compared to control tumors (arrows, Figure 4A). The number of tumors displaying invasion into surrounding tissues, including fibroadipose tissue, muscle, mammary duct, and dermis, was significantly lower in shRNA than control tumors (*P* < 0.05; Figure 4B and Supplementary Figure S6A, S6B). The *in vitro* invasiveness of shRNA cells was not further reduced by TTX (30 μM), suggesting that the impaired invasion of these cells was specifically due to the absence of Na_v_1.5 activity (Figure 4C). Furthermore, the density of cells expressing matrix metalloproteinase-9 (MMP9), a marker correlating with invasive capacity in carcinomas [23, 32], was also significantly reduced by 61% in shRNA tumors compared to control tumors (*P* < 0.05; Figure 4D, 4E). The MMP9 immunoreactivity was generally adjacent to human nuclear antigen (HNA)-positive cells, suggesting that it is being expressed by tumor cells, rather than by HNA-negative murine host stromal cells (Supplementary Figure S7). Thus, Na_v_1.5 promotes tumor growth and local invasion into surrounding tissue *in vivo*.

**Figure 5:**
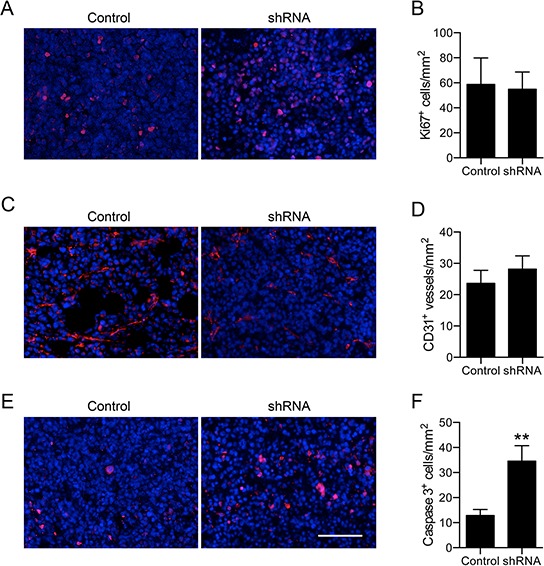
Effect of Na_v_1.5 on proliferation, apoptosis and angiogenesis **A.** Tumor stained with anti-Ki67 (red) and DAPI (blue). **B.** Ki67^+^ nuclei/mm^2^ (*n* = 7). **C.** Blood vessels stained with anti-CD31 (red) and DAPI (blue). **D.** CD31^+^ blood vessels/mm^2^ (*n* = 7). **E.** Tumor stained with anti-activated caspase-3 (red) and DAPI (blue). **F.** Activated caspase-3^+^ cells/mm^2^ (*n* = 7). Data are mean and SEM; **P < 0.01. Scale bar, 100 μm.

There was no difference in the density of Ki67^+^ dividing cells between control and shRNA tumors (Figure 5A, 5B). Similarly, there was no difference in the density of CD31-expressing vascular structures between control and shRNA tumors (Figure 5C, 5D). However, the density of activated caspase 3^+^ apoptotic cells was significantly increased by 2.7-fold in shRNA tumors compared to control (*P* < 0.01; Figure 5E, 5F). The shRNA had no effect on apoptosis in the same cells cultured *in vitro* (Supplementary Figure S3B), suggesting that Na_v_1.5/Na^+^ current may regulate apoptosis *in vivo*, but not *in vitro*. Interestingly, β1 over-expression also reduces the density of apoptotic cells in orthotopic tumors [22]. Thus, both Na_v_1.5 and β1 may increase tumor growth by reducing apoptosis.

### Na_v_1.5 down-regulation inhibits metastasis

**Figure 6:**
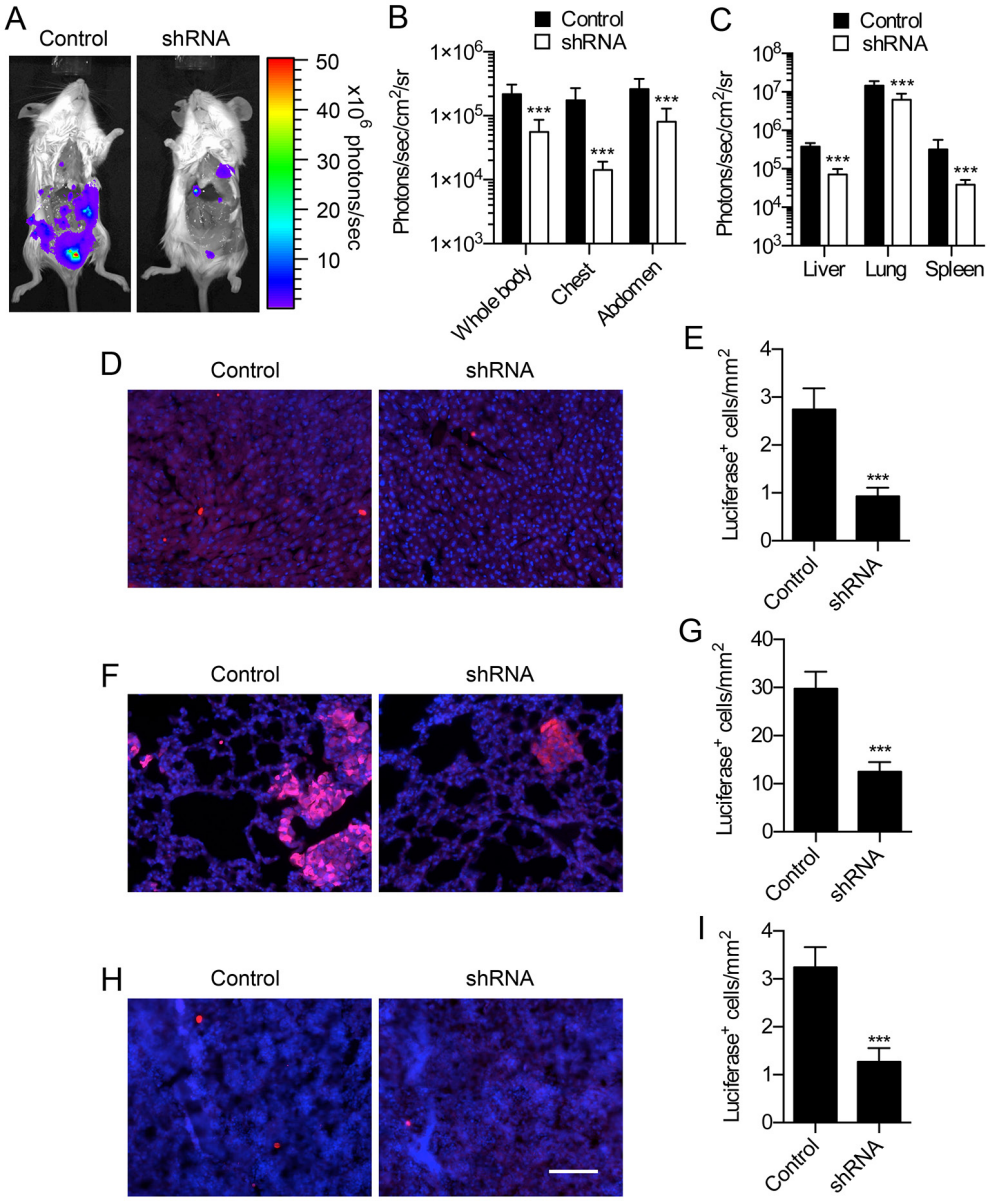
Effect of Na_v_1.5 on breast cancer metastasis **A.** Bioluminescent images of metastases in mice bearing tumors containing MDA-MB-231 cells stably expressing control shRNA (“Control”) and MDA-MB-231 cells expressing shRNA1 (“shRNA”). **B.** Bioluminescence measured from the indicated anatomical sites (n ≥ 12). **C.** Bioluminescence measured ex vivo from the liver, lungs and spleen (n ≥ 12). Liver **D.** lungs **F.** and spleen **H.** stained with anti-luciferase (red) and DAPI (blue). **E, G, I.** luciferase^+^ cells/mm^2^ at each site (*n* ≥ 20). Data are mean and SEM; ***P < 0.001. For (B) and (C), *P* < 0.001 between control and shRNA (two-way ANOVA). Scale bar, 100 μm.

We have previously shown that phenytoin reduces metastasis of orthotopically implanted MDA-MB-231 cells to the liver, lungs and spleen [23]. Similarly, Na_v_1.5 inhibition by ranolazine has recently been shown to inhibit lung colonization by tail vein-injected MDA-MB-231 cells in an experimental metastasis model [31]. Thus, pharmacological inhibition of Na_v_1.5 may be an effective tool to reduce metastatic dissemination. However, the specific effect of Na_v_1.5 on metastasis of orthotopically implanted tumors has not been investigated. Here, we monitored metastasis by bioluminescent imaging following *post mortem* resection of orthotopically implanted control and shRNA-expressing MDA-MB-231 cells (Figure 6A). We found that photon flux was significantly reduced in mice bearing shRNA tumors compared to mice bearing control tumors, and in the liver, lungs and spleen measured *ex vivo* (*P* < 0.001 for both; Figure 6B, 6C). We next studied metastasis to these organs at the cellular level in tissue sections. We detected isolated luciferase-expressing tumor cells in sections within all three organs, and also in much larger multicellular foci in the lungs Figure 6D, 6F, 6H). Luciferase expression co-localized with HNA, which is absent in recipient mouse cells, thus confirming that luciferase expression was retained on the tumor cells at metastatic sites (Supplementary Figure S8). The density of luciferase-expressing tumor cells was significantly reduced in the liver, lungs and spleen of mice bearing shRNA tumors by ~60% compared to mice bearing control tumors (*P* < 0.001; Figure 6E, 6G, 6I). Thus, Na_v_1.5 promotes metastasis to the liver, lungs and spleen in this orthotopic tumor model.

### Na_v_1.5 down-regulation alters cellular morphology and reduces CD44 expression without affecting the epithelial-mesenchymal transition

**Figure 7:**
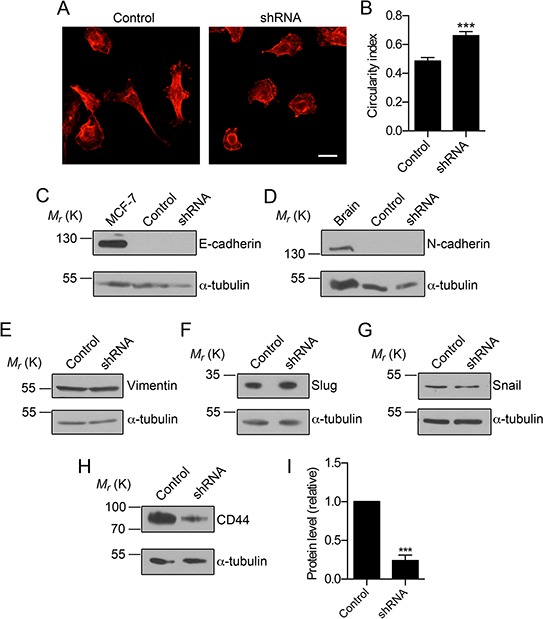
Na_v_1.5 regulates CD44, but not EMT marker expression **A.** Images of MDA-MB-231 cells stably expressing control shRNA (“Control”) and MDA-MB-231 cells expressing shRNA1 (“shRNA”), stained with anti-CD44 (red). Scale bar, 20 μm. **B.** Circularity index of control and shRNA cells (*n* ≥ 34). **C.** Western blot of E-cadherin in control and shRNA cells. Positive control = MCF-7 cells. **D.** Western blot of N-cadherin in control and shRNA cells. Positive control = rat brain. **E.** Western blot of vimentin in control and shRNA cells. **F.** Western blot of slug in control and shRNA cells. **G.** Western blot of snail in control and shRNA cells. **H.** Western blot of CD44 in in control and shRNA cells. **I.** CD44 protein levels determined by densitometry (*n* = 5). Loading control for densitometry, α-tubulin. Data are mean and SEM; ***P < 0.001.

β1 regulates protrusion of processes from the cell body of BCa cells *via* a *trans*-homophilic adhesion mechanism that requires fyn kinase and Na^+^ current, replicating its role in regulating neurite outgrowth in the CNS [22]. Similarly, Na^+^ current carried by Na_v_1.5 promotes pro-invasive, elongate morphology in MDA-MB-231 cells cultured on Matrigel [19, 31]. Thus, VGSC activity may represent a general mechanism by which BCa cells acquire an elongate, mesenchymal-like morphology. Here, we found that down-regulation of Na_v_1.5 expression with shRNA resulted in an increase in circularity of cultured MDA-MB-231 cells, i.e. a reduction in elongate morphology in favor of a more rounded epithelial-like phenotype (*P* < 0.001; Figure 7A, 7B). The reversion to a more epithelial-like morphology induced by Na_v_1.5 down-regulation did not associate with any alteration in E-cadherin, N-cadherin, vimentin, slug or snail expression, suggesting that Na_v_1.5 does not regulate the expression of epithelial-mesenchymal transition (EMT) markers (Figure 7C–7G). However, the protein level of CD44, which promotes invasion and metastasis of BCa cells [33], was noticeably reduced in MDA-MB-231 cells expressing shRNA (*P* < 0.05 Figure 7H, 7I). Thus, Na_v_1.5 may promote invasion and metastasis, at least in part, by inducing morphological changes *via* modulating CD44 expression.

## DISCUSSION

Increasing evidence suggests that VGSCs are expressed in cells from a range of types of cancer, including BCa [13, 14], cervical cancer [34, 35], colorectal cancer [21], lung cancer [25, 36], lymphoma [37], melanoma [38], mesothelioma [39], neuroblastoma [40], ovarian cancer [41] and prostate cancer [29]. VGSC β subunits have also been reported in BCa, non-small cell lung cancer and prostate cancer [22, 24, 25, 42, 43]. In BCa cells, the predominant α subunit, Na_v_1.5, is expressed mainly in its neonatal splice form [14]. A small study of patient tissue specimens revealed that the neonatal splice variant of Na_v_1.5 is up-regulated in BCa compared to non-cancer breast tissue [14]. Here, in a quantitative study using a larger patient cohort, we found that Na_v_1.5 was significantly up-regulated in breast tumors compared with matched non-cancer breast tissue. The commercially available antibody used here recognizes both the adult and neonatal splice Na_v_1.5 variants, suggesting that although the neonatal splice variant is predominant [14], splice variant specificity is not required to reliably evaluate Na_v_1.5 expression in tumors *in vivo*. Interestingly, this antibody has also been also used to demonstrate up-regulation of Na_v_1.5 expression in colorectal tumor specimens [21]. At the mRNA level, *SCN5A* expression has been shown to correlate with metastasis, recurrence and reduced overall survival in BCa patients [14, 16]. However, there was no correlation between Na_v_1.5 protein expression and other histopathological characteristics in this cohort, although there was a moderate trend towards higher Na_v_1.5 expression in node-positive patients. In conclusion, our data add support to the notion that Na_v_1.5 is up-regulated in BCa and suggest that further, larger scale studies are warranted to explore the relationship between Na_v_1.5 expression and other tumor parameters, including lymph node metastasis.

Na^+^ currents have been recorded from a number of metastatic cancer cell lines and cancer cells isolated from tumor biopsies *in vitro* using whole-cell patch clamp recording, suggesting that VGSCs may be functionally active in tumors [4, 34, 35]. In addition, pharmacological and siRNA studies have shown that Na_v_1.5 expression in metastatic MDA-MB-231 cells promotes several cellular behaviors associated with metastasis, including migration, galvanotaxis, detachment from substrates, and invasion [13, 14, 17, 44]. Similar results have been reported in cells from other tumor types, suggesting that contribution of VGSCs to migration/invasion of cancer cells may be a general phenomenon [4]. However, evidence supporting the specific involvement of Na_v_1.5 in tumor progression *in vivo* is limited. Using tissue slice recording, we found that functional Na^+^ currents were retained in orthotopic tumors. Interestingly, the Na^+^ current density was broadly similar in cells at the tumor periphery compared to those deeper into the tumor slice, suggesting that Na_v_1.5 expression may be fairly uniform within tumors, rather than being up-regulated at the invasive edge. These data provide the first direct *in vivo* evidence confirming functional VGSC activity/Na^+^ current in BCa cells tumors *in situ*, and suggest that Na_v_1.5 expression detected by IHC in patient specimens may represent, at least in part, functional channels. Thus, we propose that Na_v_1.5 should be further investigated as a potential biomarker in BCa, and *in vivo* electrophysiological analysis may add value to traditional IHC/PCR approaches to studying biomarker expression.

We found that stable down-regulation of Na_v_1.5 with shRNA reduced tumor growth *in vivo*. In addition, shRNA slightly reduced proliferation *in vitro*, although Ki67 expression was unchanged *in vivo*, suggesting that proliferation was not significantly altered in the tumors. In contrast, shRNA had no effect on apoptosis *in vitro*, but significantly increased density of apoptotic cells within the tumors. Interestingly, transient inhibition of Na_v_1.5 with TTX or phenytoin does not affect proliferation *in vitro*, although the effect on apoptosis has not been previously investigated [13, 14, 16]. Thus, the effect of Na_v_1.5 on proliferation and apoptosis appears to be both subtle and complex, and may be dependent on cellular context. For example, heterotypic interaction with, and support from the tumor microenvironment may be important for regulating Na_v_1.5-dependent tumor growth. Interestingly, we previously reported a similar situation for β1, which promotes tumor growth *in vivo*, but not *in vitro*, highlighting the importance of studying VGSC expression/activity *in situ* [22].

**Figure 8:**
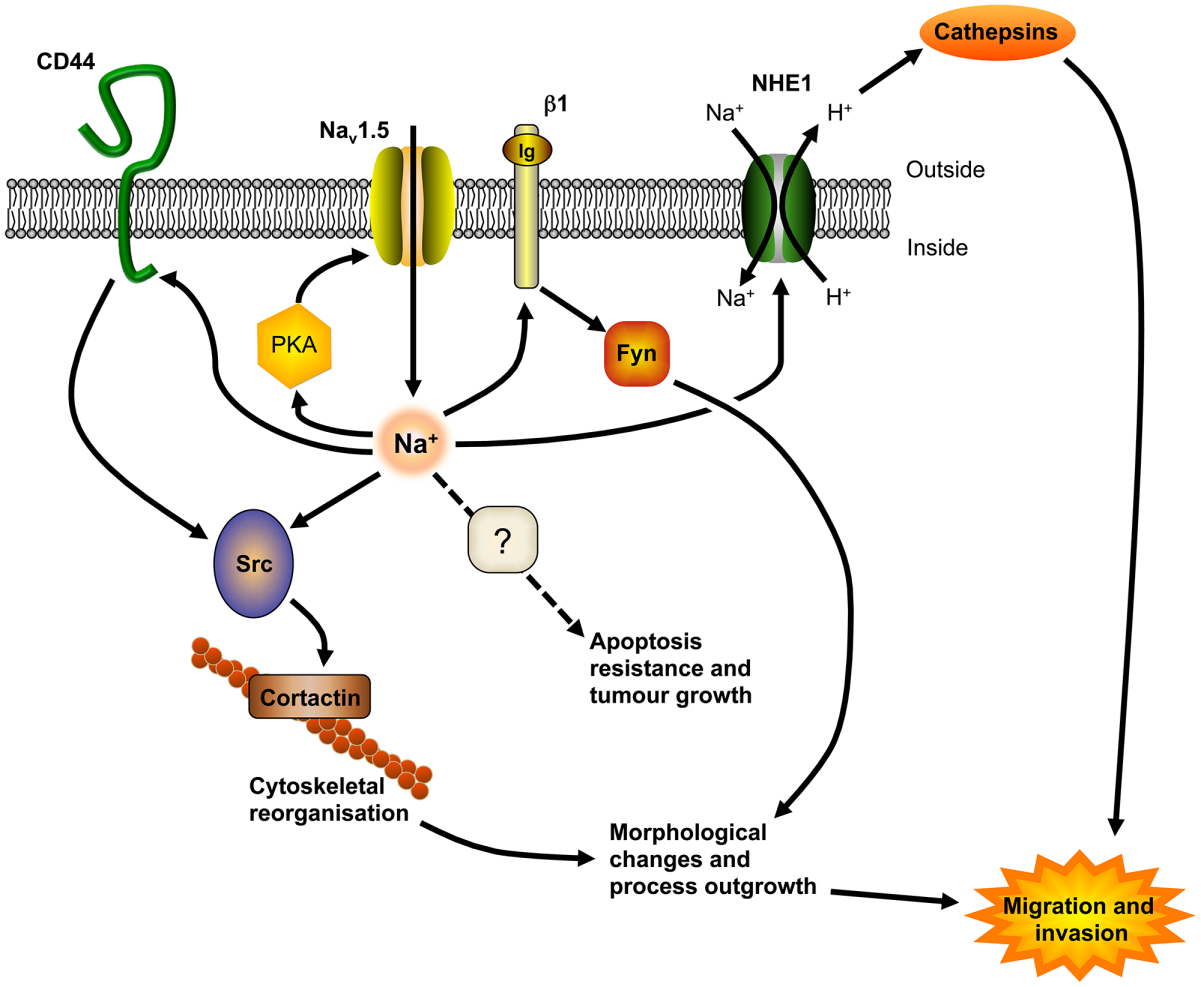
A model for Na_v_1.5 involvement in tumor progression Na^+^ influx carried by Na_v_1.5 allosterically regulates NHE1 to increase H^+^ efflux, lowering extracellular pH and enhancing cathepsin protease activity [18, 20]. Na_v_1.5 also increases src activity and cortactin phosphorylation, resulting in cytoskeletal changes, invadopodia formation and acquisition of an elongate invasive morphology [19]. The β1 subunit enhances process outgrowth *via* a *trans*-homophilic adhesion mechanism that requires fyn kinase and Na^+^ current [22]. Na_v_1.5 positively regulates CD44 protein expression, which may in turn enhance CD44-mediated src activation and increase cortactin expression/activity [47–49]. Na_v_1.5 expression is maintained by a positive feedback mechanism *via* protein kinase A (PKA) [26, 57]. Additional potential partners in this signaling network have been omitted for clarity, and are reviewed in detail elsewhere [4, 45]. Figure was produced using ScienceSlides software.

Our data suggest that Na_v_1.5 promotes local invasion and metastatic dissemination to the liver, lungs and spleen *in vivo*. This finding broadly agrees with a recent study, using an experimental metastasis model, in which Na_v_1.5 down-regulation reduced lung colonization of tail vein-injected MDA-MB-231 cells [31]. Together, these findings suggest that Na_v_1.5 may promote both tumor growth and metastasis in BCa. The mechanism(s) by which VGSCs may promote tumor progression appear complex, and are reviewed in detail elsewhere [4, 45]. The prevailing model suggests that in MDA-MB-231 cells, Na^+^ current carried by Na_v_1.5 allosterically regulates NHE1 to increase H^+^ efflux, thus enhancing the activity of pH-dependent cysteine cathepsin proteases [18, 20]. In addition, Na_v_1.5 expression increases src tyrosine kinase activity and cortactin phosphorylation to enhance invadopodia formation and acquisition of an elongate invasive morphology [19]. Further complexity is added by the β1 subunit, which also promotes an invasive elongate cellular morphology, in part *via* a *trans*-homophilic adhesion interaction that requires fyn kinase [22]. Interestingly, β1-mediated process outgrowth also requires Na^+^ current, suggesting that Na_v_1.5 and β1 may function co-operatively in promoting invasion and metastasis. Our data here add to this model by suggesting that Na_v_1.5 expression does not enhance invasive elongate cellular morphology by altering the EMT, but may directly/indirectly influence expression of the metastasis-promoting CAM, CD44 (Figure 8) [33, 46]. Adhesion of CD44 to its ligand hyaluronan results in src activation and cortactin phosphorylation [47–49]. Thus, Na_v_1.5 may either directly/indirectly regulate this signaling cascade, or regulate cortactin phosphorylation through a parallel pathway.

In conclusion, our data suggest that Na_v_1.5 is up-regulated in BCa and promotes both tumor growth and metastasis. Thus, VGSC α and β subunits may be general mediators of an invasive phenotype in tumor cells. Given that pharmacological targeting of VGSCs inhibits tumor growth and metastasis *in vivo* [23, 30, 31], these findings suggest that Na_v_1.5 expression and/or Na^+^ current may be a useful biomarker for cancer progression, and VGSC inhibition may be a novel therapeutic strategy to reduce metastasis.

## MATERIALS AND METHODS

### Ethics statement

Investigation has been conducted in accordance with the ethical standards according to the Declaration of Helsinki and according to national and international guidelines and has been approved by the University of York Ethical Review Process.

### Cell culture

Generation of MDA-MB-231 cells stably expressing eGFP and firefly luciferase was described previously [22]. MCF-7 cells were a gift from M. Djamgoz, Imperial College London. BT474 and SKBR3 cells were a gift from J. Rae, University of Michigan. MCF-10A cells were a gift from N. Maitland, University of York. HEK293 cells stably expressing Na_v_1.5 were a gift from L. Isom, University of Michigan. Cell lines were grown in Dulbecco's modified eagle medium (DMEM) supplemented with 5% FBS and 4 mM L-glutamine. Molecular identity of cells was confirmed by short tandem repeat analysis. Cells were confirmed as mycoplasma-free using the DAPI method.

### Pharmacology and *in vitro* assays

TTX was diluted in culture medium to 30 μM. Staurosporine was prepared as stock in DMSO and then diluted in culture medium. In assays that exceeded 24 h, treatments were replaced daily. *In vitro* migration and invasion were determined using wound healing and Matrigel assays [16]. Apoptosis was determined using DeadEnd fluorometric TUNEL assay (Promega). Cell viability and proliferation were determined using trypan blue and MTT assays [16].

### RNA interference, RNA isolation and PCR

MDA-MB-231 cells stably expressing eGFP and luciferase were stably transduced with recombinant lentivirus for one of four different shRNAs targeting Na_v_1.5 or a non-targeting control shRNA, according to the manufacturer's instructions (MISSION pLKO.1-puro shRNA transduction particles; Sigma). Sequences are in Supplementary Table S2. ShRNA-expressing cells were selected using resistance to puromycin and individual colonies were expanded for screening by qPCR. RNA extraction, cDNA synthesis and qPCR were performed as described previously [22]. Relative gene expression was quantitated using the comparative C_T_ method [50]. Primers are in Supplementary Table S3.

### Patient tissue samples

The study cohort contained tissue samples from 66 BCa cases obtained from the Breast Cancer Now Tissue Bank under tissue request number TR000032. BCa and matched non-cancer breast tissue was available for 36 cases. Patients provided consent to the Breast Cancer Now Tissue Bank for their tissues to be used for research. Dataset and sample characteristics were described previously [22]. Immunohistochemistry was performed using the EnVision+ System-HRP kit (Dako) and anti-Na_v_1.5 antibody (1:100; Alomone) [21, 22]. Antibody specificity was confirmed by preadsorption to immunizing peptide (4:1 ratio peptide:antibody; Alomone) for 1 h prior to application. Rat heart tissue (Abcam) was used as a positive control for Na_v_1.5 staining in a separate experiment (Supplementary Figure S1). β1 immunohistochemistry was performed previously [22]. Slides were scanned at 40X using an Aperio ScanScope. Na_v_1.5 immunoreactivity in the tumor samples was scored independently by two investigators (WJB and RMS, a breast histopathologist) using the Allred method [22, 51]. The proportion of Na_v_1.5-expressing cells in a given section was given a score (none: 0; < 1/100: 1; 1/100 to 1/10: 2; 1/10 to 1/3: 3; 1/3 to 2/3: 4; >2/3: 5), followed by an estimate of staining intensity (none: 0; weak: 1; intermediate: 2; strong: 3), and then the proportion and intensity scores were summed to give an overall score of 0–8. A score ≤ 5 was considered “low” and > 5 was “high.” Scoring was performed blinded to outcome data.

### Western blotting

SDS-PAGE was performed as described [22]. The following antibodies were used: rabbit anti-Na_v_1.5 (1:1000; Cell Signaling Technology); rabbit anti-E-cadherin (1:1000; Cell Signaling Technology); rabbit anti-N-cadherin (1:1000; Cell Signaling Technology); rabbit anti-vimentin (1:1000; Cell Signaling Technology); rabbit anti-Slug (1:1000; Cell Signaling Technology); rabbit anti-Snail (1:1000; Cell Signaling Technology); mouse anti-CD44 (1:1000; AbD Serotec); rabbit anti-heat shock protein 90 (HSP90; 1:1000; Cell Signaling Technology), and mouse anti-α-tubulin (1:10,000; Sigma).

### Orthotopic breast tumor model

All animal procedures were performed after approval by the University of York Animal Welfare and Ethical Review Body and under authority of a UK Home Office Project License. Six week-old female *Rag2*^−/−^ *Il2rg*^−/−^ mice were obtained from the Yorkshire Cancer Research Unit, University of York, (3–5 per specific pathogen free cage). 1 × 10^6^ MDA-MB-231 cells suspended in Matrigel (20% v/v in saline) were injected into the left inguinal mammary fat pad of each animal whilst under isoflurane anesthesia. For tumor growth and metastasis assays, a total of 29 mice were used across 7 independent replicated experiments. Tumor growth was monitored weekly by bioluminescence imaging [22]. Animal weight and the length and width of each tumor (in mm) were measured every 2–4 days. Tumor volume was calculated as 0.5 × (length × width^2^). Mice were euthanized 4 weeks following implantation of tumor cells and metastatic bioluminescence was measured [22]. Tumors and organ sites of metastasis were fixed in 4% paraformaldehyde and frozen [52].

### Tumor slice preparation for electrophysiology

For electrophysiological slice recording, a total of 18 animals were used across 6 independent cages. Following euthanasia (20–37 days following implantation of cancer cells), tumors were dissected and quickly placed into ice-cold physiological saline solution (PSS) containing (in mM): 144 NaCl, 5.4 KCl, 1 MgCl_2_, 2.5 CaCl_2_, 5.6 D-glucose and 5 HEPES, (pH 7.2). The tumor was cut to ~5 × 5 mm with a razor and fixed with cyanoacrylate glue onto the pre-chilled pedestal of an oscillating tissue slicer (Campden Instruments). The tissue block was immersed in ice-cold PSS and sliced at 250 μm. Slices were maintained in a homemade tissue holding chamber containing PSS for > 20 min at room temperature (21°C) prior to recording. In all cases, recordings were taken from cells in (1) the periphery (≤1 mm from tumor surface, (2) the intermediate zone (>1 mm and ≤ 1.5 mm from surface), and (3) the center (>1.5 mm from surface).

### Electrophysiology

The whole-cell patch clamp technique was used to record plasma membrane Na^+^ currents, from cells in slices, or cells grown on glass coverslips [16, 24]. The extracellular recording solution contained (in mM): 144 NaCl, 5.4 KCl, 1 MgCl_2_, 2.5 CaCl_2_, 5.6 D-glucose and 5 HEPES (pH 7.2), and the intracellular recording solution contained (in mM): 5 NaCl, 145 KCl, 2 MgCl_2_, 1 CaCl_2_, 10 HEPES, 11 EGTA, (pH 7.4) [26]. Voltage clamp recordings were made using a Multiclamp 700B amplifier (Molecular Devices) compensating for series resistance by 40–60%. Currents were digitized using a Digidata 1440A interface (Molecular Devices), low-pass filtered at 10 kHz, sampled at 50 kHz and analyzed using pCLAMP 10.4 software (Molecular Devices). Leak current was subtracted using a P/6 protocol [53].

### Immunohistochemistry and immunocytochemistry

H&E staining was performed as described [22]. The following primary antibodies were used for IHC and immunocytochemistry (ICC) [22]: rabbit anti-MMP9 (1:5000; Abcam); rabbit anti-Ki67 (1:5000; Abcam); rabbit anti-activated caspase-3 (1:200; R&D Systems); rabbit anti-CD31 (Santa Cruz Biotechnology); mouse anti-CD44 (1:100; AbD Serotec); mouse anti-HNA (1:100; Millipore). Secondary antibodies were Alexa-568-conjugated goat anti mouse/rabbit, unless stated otherwise (1:500; Invitrogen). Tyramide signal amplification was used for MMP9 [54]. Samples were mounted in Prolong Gold with DAPI (Invitrogen). H&E and fluorescent-stained tissue sections were scanned independently at 20X using a Zeiss AxioScan.Z1 slide scanner. Stained cultured cells were viewed on a Zeiss Axio Observer.Z1 microscope with LSM 710 confocal laser scanner. Images were exported into ImageJ for processing. Brightness/contrast was adjusted using the ImageJ “Auto” function. Density of MMP9^+^, Ki67^+^ or activated caspase-3^+^ cells, tumor vascularity and metastasis to liver/lungs/spleen were measured across scanned images of whole sections (3 sections per animal), blinded to treatment [22]. For ICC, confocal Z-series projections were flattened using the maximum signal and circularity ([4π.Area]/[Perimeter^2^]) was computed for individual cells using ImageJ. For a perfect circle, circularity = 1, and for an increasingly elongated shape, circularity approaches 0 [55].

### Data analysis

Data are mean and SEM unless stated otherwise. Statistical analysis was performed using GraphPad Prism 6f. Pairwise statistical significance was determined with *t*-tests. Multiple comparisons were made using ANOVA and Tukey post-hoc tests, unless stated otherwise. TUNEL assay and tumor local invasion data were analyzed by two-way ANOVA. Metastatic bioluminescence data were log-transformed and analyzed by two-way ANOVA. Correlation was determined using Pearson's r test. Association between Na_v_1.5 expression and histoclinical data was determined using Fisher's exact or χ^2^ tests. Results were considered significant at *P* < 0.05.

## SUPPLEMENTARY FIGURES AND TABLES



## References

[1] Autier P, Boniol M, La Vecchia C, Vatten L, Gavin A, Hery C, Heanue M (2010). Disparities in breast cancer mortality trends between 30 European countries: retrospective trend analysis of WHO mortality database. BMJ.

[2] Jemal A, Bray F, Center MM, Ferlay J, Ward E, Forman D (2011). Global cancer statistics. CA Cancer J Clin.

[3] Eccles SA, Aboagye EO, Ali S, Anderson AS, Armes J, Berditchevski F, Blaydes JP, Brennan K, Brown NJ, Bryant HE, Bundred NJ, Burchell JM, Campbell AM, Carroll JS, Clarke RB, Coles CE (2013). Critical research gaps and translational priorities for the successful prevention and treatment of breast cancer. Breast Cancer Res.

[4] Brackenbury WJ (2012). Voltage-gated sodium channels and metastatic disease. Channels (Austin).

[5] Djamgoz MB, Coombes RC, Schwab A (2014). Ion transport and cancer: from initiation to metastasis. Philos Trans R Soc Lond B Biol Sci.

[6] Brackenbury WJ, Isom LL (2011). Na Channel beta Subunits: Overachievers of the Ion Channel Family. Front Pharmacol.

[7] Mantegazza M, Curia G, Biagini G, Ragsdale DS, Avoli M (2010). Voltage-gated sodium channels as therapeutic targets in epilepsy and other neurological disorders. Lancet Neurol.

[8] Brackenbury WJ, Calhoun JD, Chen C, Miyazaki H, Nukina N, Oyama F, Ranscht B, Isom LL (2010). Functional reciprocity between Na+ channel Nav1.6 and β1 subunits in the coordinated regulation of excitability and neurite outgrowth. Proc Natl Acad Sci U S A.

[9] Chopra SS, Stroud DM, Watanabe H, Bennett JS, Burns CG, Wells KS, Yang T, Zhong TP, Roden DM (2010). Voltage-gated sodium channels are required for heart development in zebrafish. Circ Res.

[10] George AL (2005). Inherited disorders of voltage-gated sodium channels. J Clin Invest.

[11] Papadatos GA, Wallerstein PM, Head CE, Ratcliff R, Brady PA, Benndorf K, Saumarez RC, Trezise AE, Huang CL, Vandenberg JI, Colledge WH, Grace AA (2002). Slowed conduction and ventricular tachycardia after targeted disruption of the cardiac sodium channel gene Scn5a. Proc Natl Acad Sci U S A.

[12] Black JA, Waxman SG (2013). Noncanonical roles of voltage-gated sodium channels. Neuron.

[13] Roger S, Besson P, Le Guennec JY (2003). Involvement of a novel fast inward sodium current in the invasion capacity of a breast cancer cell line. Biochim Biophys Acta.

[14] Fraser SP, Diss JK, Chioni AM, Mycielska ME, Pan H, Yamaci RF, Pani F, Siwy Z, Krasowska M, Grzywna Z, Brackenbury WJ, Theodorou D, Koyuturk M, Kaya H, Battaloglu E, De Bella MT (2005). Voltage-gated sodium channel expression and potentiation of human breast cancer metastasis. Clin Cancer Res.

[15] Brackenbury WJ, Djamgoz MB, Isom LL (2008). An emerging role for voltage-gated Na+ channels in cellular migration: regulation of central nervous system development and potentiation of invasive cancers. Neuroscientist.

[16] Yang M, Kozminski DJ, Wold LA, Modak R, Calhoun JD, Isom LL, Brackenbury WJ (2012). Therapeutic potential for phenytoin: targeting Na(v)1.5 sodium channels to reduce migration and invasion in metastatic breast cancer. Breast Cancer Res Treat.

[17] Brackenbury WJ, Chioni AM, Diss JK, Djamgoz MB (2007). The neonatal splice variant of Nav1.5 potentiates *in vitro* metastatic behaviour of MDA-MB-231 human breast cancer cells. Breast Cancer Res Treat.

[18] Gillet L, Roger S, Besson P, Lecaille F, Gore J, Bougnoux P, Lalmanach G, Le Guennec JY (2009). Voltage-gated Sodium Channel Activity Promotes Cysteine Cathepsin-dependent Invasiveness and Colony Growth of Human Cancer Cells. J Biol Chem.

[19] Brisson L, Driffort V, Benoist L, Poet M, Counillon L, Antelmi E, Rubino R, Besson P, Labbal F, Chevalier S, Reshkin SJ, Gore J, Roger S (2013). NaV1.5 Na(+) channels allosterically regulate the NHE-1 exchanger and promote the activity of breast cancer cell invadopodia. J Cell Sci.

[20] Brisson L, Gillet L, Calaghan S, Besson P, Le Guennec JY, Roger S, Gore J (2011). Na(V)1.5 enhances breast cancer cell invasiveness by increasing NHE1-dependent H(+) efflux in caveolae. Oncogene.

[21] House CD, Vaske CJ, Schwartz A, Obias V, Frank B, Luu T, Sarvazyan N, Irby RB, Strausberg RL, Hales T, Stuart J, Lee NH (2010). Voltage-gated Na+ channel SCN5A is a key regulator of a gene transcriptional network that controls colon cancer invasion. Cancer Res.

[22] Nelson M, Millican-Slater R, Forrest LC, Brackenbury WJ (2014). The sodium channel beta1 subunit mediates outgrowth of neurite-like processes on breast cancer cells and promotes tumour growth and metastasis. Int J Cancer.

[23] Nelson M, Yang M, Dowle AA, Thomas JR, Brackenbury WJ (2015). The sodium channel-blocking antiepileptic drug phenytoin inhibits breast tumour growth and metastasis. Mol Cancer.

[24] Chioni AM, Brackenbury WJ, Calhoun JD, Isom LL, Djamgoz MB (2009). A novel adhesion molecule in human breast cancer cells: voltage-gated Na+ channel beta1 subunit. Int J Biochem Cell Biol.

[25] Roger S, Rollin J, Barascu A, Besson P, Raynal PI, Iochmann S, Lei M, Bougnoux P, Gruel Y, Le Guennec JY (2007). Voltage-gated sodium channels potentiate the invasive capacities of human non-small-cell lung cancer cell lines. Int J Biochem Cell Biol.

[26] Brackenbury WJ, Djamgoz MB (2006). Activity-dependent regulation of voltage-gated Na+ channel expression in Mat-LyLu rat prostate cancer cell line. J Physiol.

[27] Brackenbury WJ, Djamgoz MB (2007). Nerve growth factor enhances voltage-gated Na+ channel activity and Transwell migration in Mat-LyLu rat prostate cancer cell line. J Cell Physiol.

[28] Ding Y, Brackenbury WJ, Onganer PU, Montano X, Porter LM, Bates LF, Djamgoz MB (2008). Epidermal growth factor upregulates motility of Mat-LyLu rat prostate cancer cells partially via voltage-gated Na+ channel activity. J Cell Physiol.

[29] Grimes JA, Fraser SP, Stephens GJ, Downing JE, Laniado ME, Foster CS, Abel PD, Djamgoz MB (1995). Differential expression of voltage-activated Na+ currents in two prostatic tumour cell lines: contribution to invasiveness *in vitro*. FEBS Lett.

[30] Yildirim S, Altun S, Gumushan H, Patel A, Djamgoz MB (2012). Voltage-gated sodium channel activity promotes prostate cancer metastasis *in vivo*. Cancer Lett.

[31] Driffort V, Gillet L, Bon E, Marionneau-Lambot S, Oullier T, Joulin V, Collin C, Pages JC, Jourdan ML, Chevalier S, Bougnoux P, Le Guennec JY, Besson P, Roger S (2014). Ranolazine inhibits NaV1.5-mediated breast cancer cell invasiveness and lung colonization. Mol Cancer.

[32] Borges S, Doppler H, Perez EA, Andorfer CA, Sun Z, Anastasiadis PZ, Thompson EA, Geiger XJ, Storz P (2013). Pharmacologic reversion of epigenetic silencing of the PRKD1 promoter blocks breast tumor cell invasion and metastasis. Breast Cancer Res.

[33] McFarlane S, Coulter JA, Tibbits P, O'Grady A, McFarlane C, Montgomery N, Hill A, McCarthy HO, Young LS, Kay EW, Isacke CM, Waugh DJ (2015). CD44 increases the efficiency of distant metastasis of breast cancer. Oncotarget.

[34] Diaz D, Delgadillo DM, Hernandez-Gallegos E, Ramirez-Dominguez ME, Hinojosa LM, Ortiz CS, Berumen J, Camacho J, Gomora JC (2007). Functional expression of voltage-gated sodium channels in primary cultures of human cervical cancer. J Cell Physiol.

[35] Hernandez-Plata E, Ortiz CS, Marquina-Castillo B, Medina-Martinez I, Alfaro A, Berumen J, Rivera M, Gomora JC (2012). Overexpression of Na(V) 1.6 channels is associated with the invasion capacity of human cervical cancer. Int J Cancer.

[36] Onganer PU, Djamgoz MB (2005). Small-cell lung cancer (human): potentiation of endocytic membrane activity by voltage-gated Na+ channel expression *in vitro*. J Membr Biol.

[37] Fraser SP, Diss JK, Lloyd LJ, Pani F, Chioni AM, George AJ, Djamgoz MB (2004). T-lymphocyte invasiveness: control by voltage-gated Na+ channel activity. FEBS Lett.

[38] Carrithers MD, Chatterjee G, Carrithers LM, Offoha R, Iheagwara U, Rahner C, Graham M, Waxman SG (2009). Regulation of podosome formation in macrophages by a novel splice variant of the sodium channel SCN8A. J Biol Chem.

[39] Fulgenzi G, Graciotti L, Faronato M, Soldovieri MV, Miceli F, Amoroso S, Annunziato L, Procopio A, Taglialatela M (2006). Human neoplastic mesothelial cells express voltage-gated sodium channels involved in cell motility. Int J Biochem Cell Biol.

[40] Ou SW, Kameyama A, Hao LY, Horiuchi M, Minobe E, Wang WY, Makita N, Kameyama M (2005). Tetrodotoxin-resistant Na+ channels in human neuroblastoma cells are encoded by new variants of Nav1.5/SCN5A. Eur J Neurosci.

[41] Gao R, Shen Y, Cai J, Lei M, Wang Z (2010). Expression of voltage-gated sodium channel alpha subunit in human ovarian cancer. Oncol Rep.

[42] Diss JK, Fraser SP, Walker MM, Patel A, Latchman DS, Djamgoz MB (2008). Beta-subunits of voltage-gated sodium channels in human prostate cancer: quantitative *in vitro* and *in vivo* analyses of mRNA expression. Prostate Cancer Prostatic Dis.

[43] Jansson KH, Lynch JE, Lepori-Bui N, Czymmek KJ, Duncan RL, Sikes RA (2012). Overexpression of the VSSC-associated CAM, beta-2, enhances LNCaP cell metastasis associated behavior. Prostate.

[44] Palmer CP, Mycielska ME, Burcu H, Osman K, Collins T, Beckerman R, Perrett R, Johnson H, Aydar E, Djamgoz MB (2008). Single cell adhesion measuring apparatus (SCAMA): application to cancer cell lines of different metastatic potential and voltage-gated Na+ channel expression. Eur Biophys J.

[45] Besson P, Driffort V, Bon E, Gradek F, Chevalier S, Roger S (2015). How do voltage-gated sodium channels enhance migration and invasiveness in cancer cells?. Biochim Biophys Acta.

[46] House CD, Wang BD, Ceniccola K, Williams R, Simaan M, Olender J, Patel V, Baptista-Hon DT, Annunziata CM, Gutkind JS, Hales TG, Lee NH (2015). Voltage-gated Na+ Channel Activity Increases Colon Cancer Transcriptional Activity and Invasion Via Persistent MAPK Signaling. Sci Rep.

[47] Bourguignon LY, Zhu H, Shao L, Chen YW (2001). CD44 interaction with c-Src kinase promotes cortactin-mediated cytoskeleton function and hyaluronic acid-dependent ovarian tumor cell migration. J Biol Chem.

[48] Turley EA, Noble PW, Bourguignon LY (2002). Signaling properties of hyaluronan receptors. J Biol Chem.

[49] Hill A, McFarlane S, Mulligan K, Gillespie H, Draffin JE, Trimble A, Ouhtit A, Johnston PG, Harkin DP, McCormick D, Waugh DJ (2006). Cortactin underpins CD44-promoted invasion and adhesion of breast cancer cells to bone marrow endothelial cells. Oncogene.

[50] Livak KJ, Schmittgen TD (2001). Analysis of relative gene expression data using real-time quantitative PCR and the 2(−Delta Delta C(T)) Method. Methods.

[51] Harvey JM, Clark GM, Osborne CK, Allred DC (1999). Estrogen receptor status by immunohistochemistry is superior to the ligand-binding assay for predicting response to adjuvant endocrine therapy in breast cancer. J Clin Oncol.

[52] Brackenbury WJ, Davis TH, Chen C, Slat EA, Detrow MJ, Dickendesher TL, Ranscht B, Isom LL (2008). Voltage-gated Na+ channel β1 subunit-mediated neurite outgrowth requires fyn kinase and contributes to central nervous system development *in vivo*. J Neurosci.

[53] Armstrong CM, Bezanilla F (1977). Inactivation of the sodium channel. II. Gating current experiments. J Gen Physiol.

[54] Brackenbury WJ, Yuan Y, O'Malley HA, Parent JM, Isom LL (2013). Abnormal neuronal patterning occurs during early postnatal brain development of Scn1b-null mice and precedes hyperexcitability. Proc Natl Acad Sci U S A.

[55] Schneider CA, Rasband WS, Eliceiri KW (2012). NIH Image to ImageJ: 25 years of image analysis. Nat Methods.

[56] Patino GA, Brackenbury WJ, Bao YY, Lopez-Santiago LF, O'Malley HA, Chen CL, Calhoun JD, Lafreniere RG, Cossette P, Rouleau GA, Isom LL (2011). Voltage-Gated Na+ Channel beta 1B: A Secreted Cell Adhesion Molecule Involved in Human Epilepsy. J Neurosci.

[57] Chioni AM, Shao D, Grose R, Djamgoz MB (2010). Protein kinase A and regulation of neonatal Nav1.5 expression in human breast cancer cells: activity-dependent positive feedback and cellular migration. Int J Biochem Cell Biol.

