# Supplementary material for: Nav1.5 regulates breast tumor growth and metastatic dissemination in vivo
Source: Oncotarget. 2015 Oct 6;6(32):32914–29. doi: 10.18632/oncotarget.5441 (PMC4741739; doi:10.18632/oncotarget.5441)
Supplement: Supplementary file 1 [file oncotarget-06-32914-s001.pdf]

SUPPLEMENTARY FIGURES AND TABLES

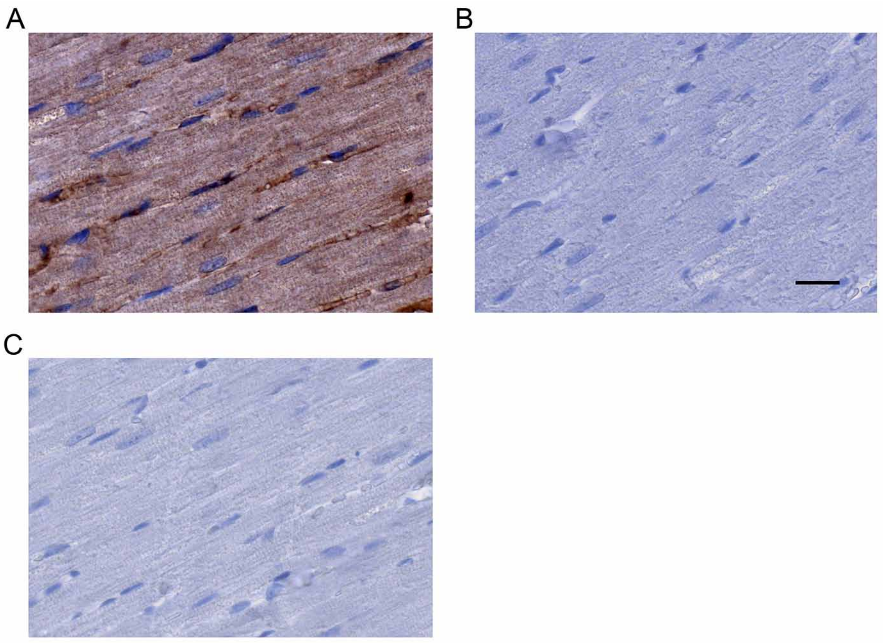

Supplementary Figure S1: Confirmation of $Na_v1.5$ antibody specificity. **A.** $Na_v1.5$ staining in rat heart. **B.** Absence of immunoreactivity in rat heart stained with anti-$Na_v1.5$ antibody preincubated with immunizing peptide. **C.** Rat heart section exposed to same staining procedure in absence of primary antibody. Scale bar, 20 µm.

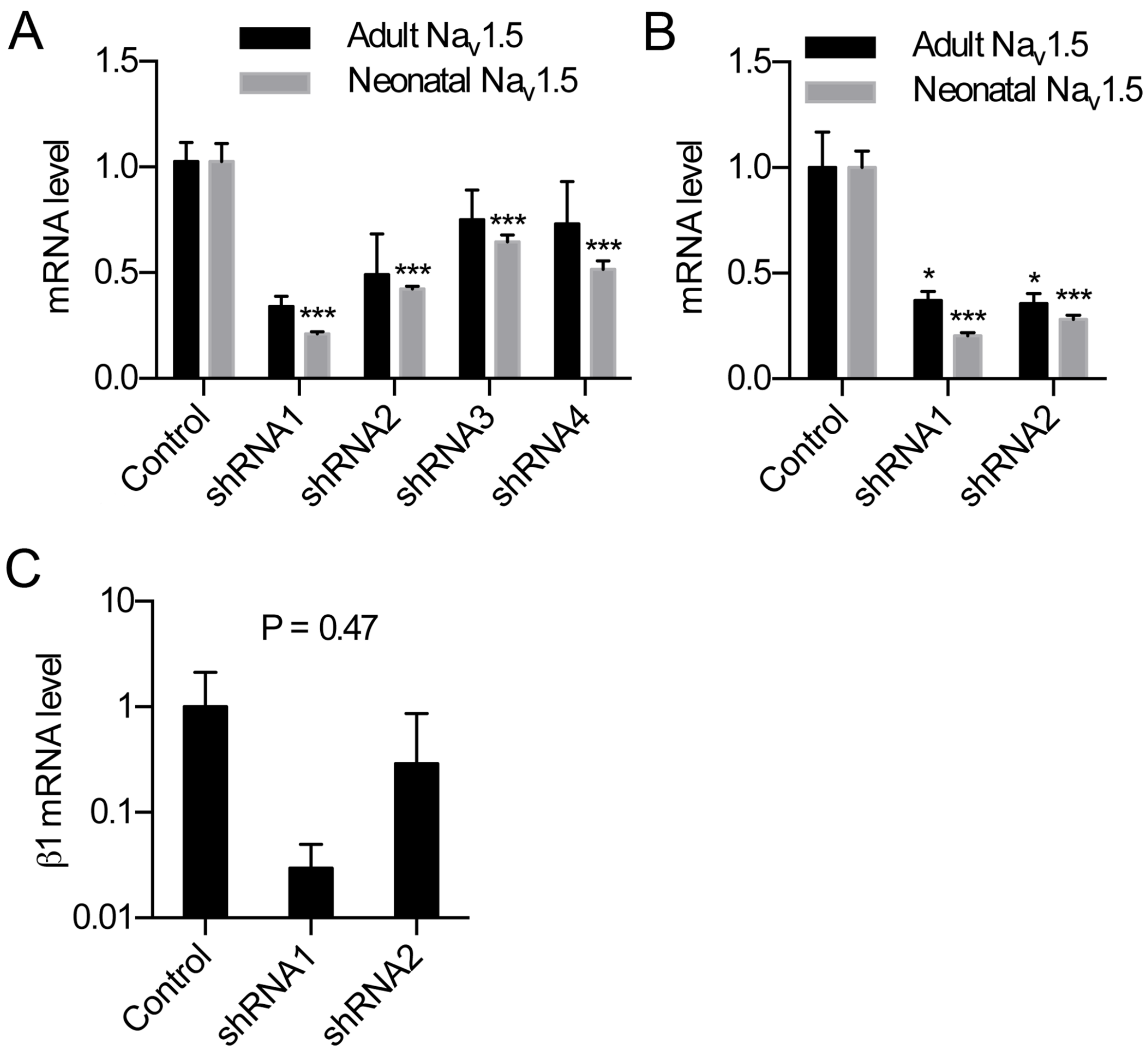

Supplementary Figure S2: Effect of shRNA on $Na_v1.5$ mRNA and protein levels. A. Expression of adult and neonatal splice variants of $Na_v1.5$ in MDA-MB-231 cells following lentiviral infection with control shRNA, or four shRNAs targeting both splice variants of $Na_v1.5$. **B.** Expression of adult and neonatal splice variants of $Na_v1.5$ in clonal populations of MDA-MB-231 cells stably expressing control shRNA, or shRNA1 or shRNA2. **C.** Expression of β1 in MDA-MB-231 cells stably expressing control shRNA, or shRNA1 or shRNA2. Data are mean and SEM. $*P < 0.05$; $***P < 0.001$.

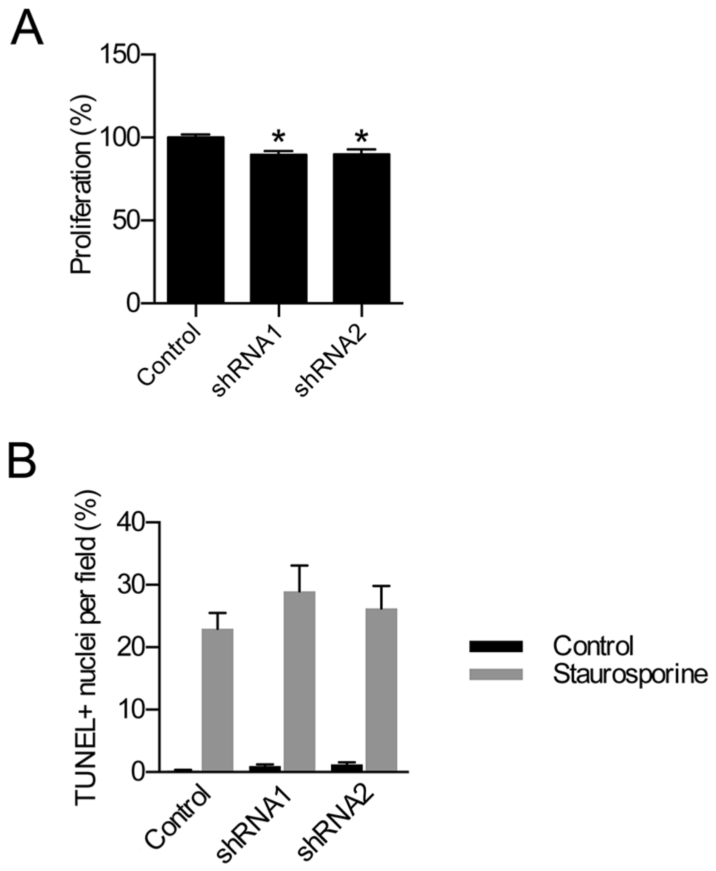

Supplementary Figure S3: Effect of Na_v1.5-targeting shRNA on proliferation and apoptosis. A. Proliferation of MDA-MB-231 cells stably expressing control shRNA, or shRNA1, or shRNA2 determined by MTT assay ($n = 9$). **B.** Proportion (%) of TUNEL-positive nuclei per field of view for MDA-MB-231 cells stably expressing control shRNA, or shRNA1, or shRNA2 treated for 24 h with/without 0.5 µM staurosporine ($n = 30$). Data are mean and SEM. *$P < 0.05$.

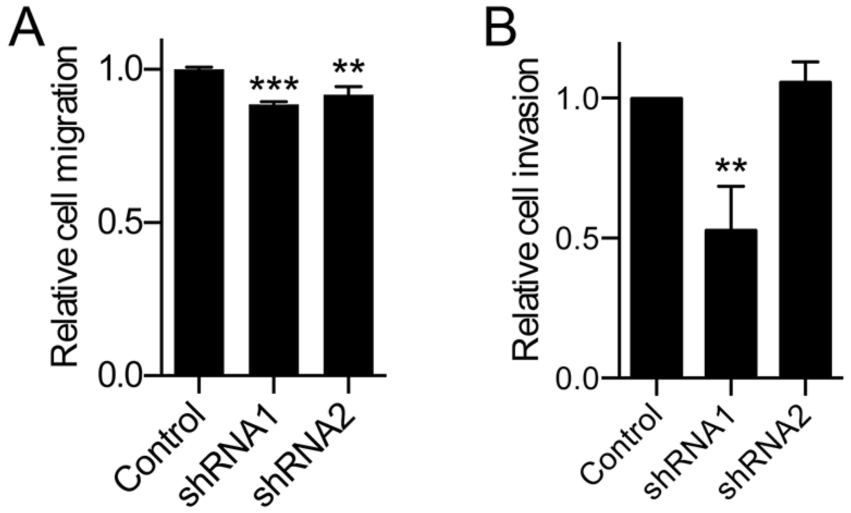

Supplementary Figure S4: Effect of Na_v1.5-targeting shRNA on migration and invasion *in vitro*. A. Migration of MDA-MB-231 cells stably expressing control shRNA, or shRNA1, or shRNA2 ($n \geq 135$). **B.** Invasion of MDA-MB-231 cells stably expressing control shRNA, or shRNA1, or shRNA2 ($n = 4$). Data are mean and SEM. **$P < 0.01$; ***$P < 0.001$.

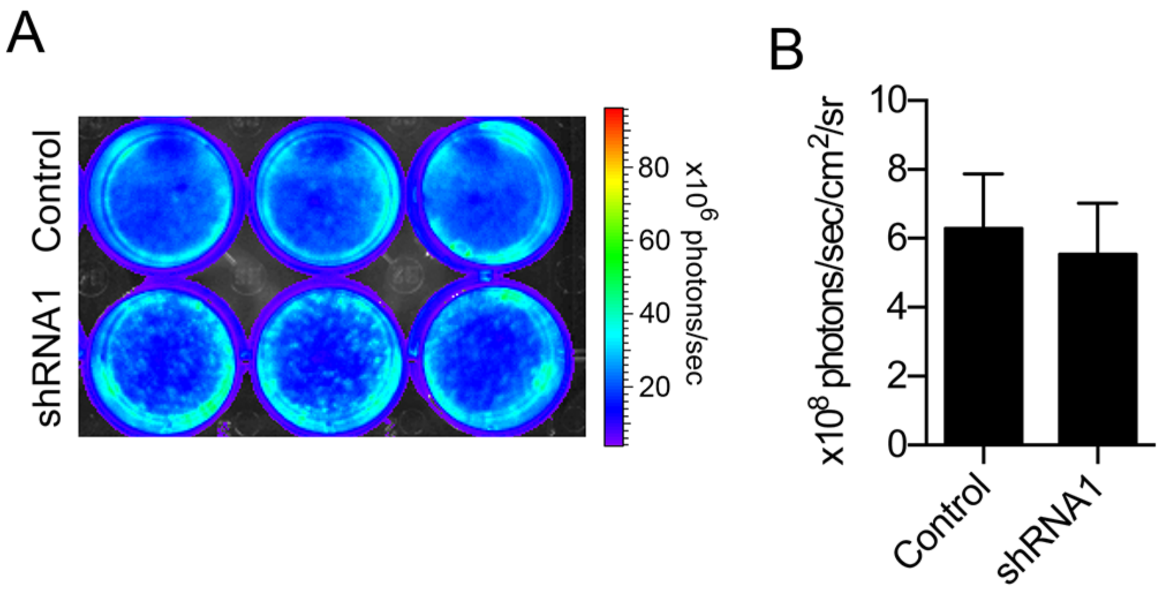

Supplementary Figure S5: Luciferase expression in MDA-MB-231 cells. A. Representative bioluminescent image of luciferase activity in cultured MDA-MB-231 cells stably expressing control shRNA or shRNA1 (1×10^5 cells/well), 10 min following addition of D-luciferin (1 mg/ml). **B.** Bioluminescence measured from cultured cells ($n = 9$). Data are mean and SEM.

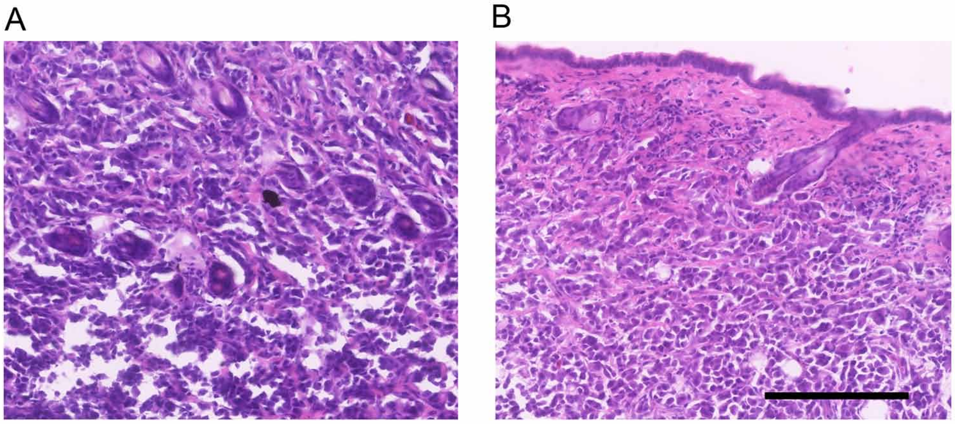

Supplementary Figure S6: Invasion of orthotopically implanted MDA-MB-231 tumour cells into duct A. and dermis B. Scale bar, 100 µm.

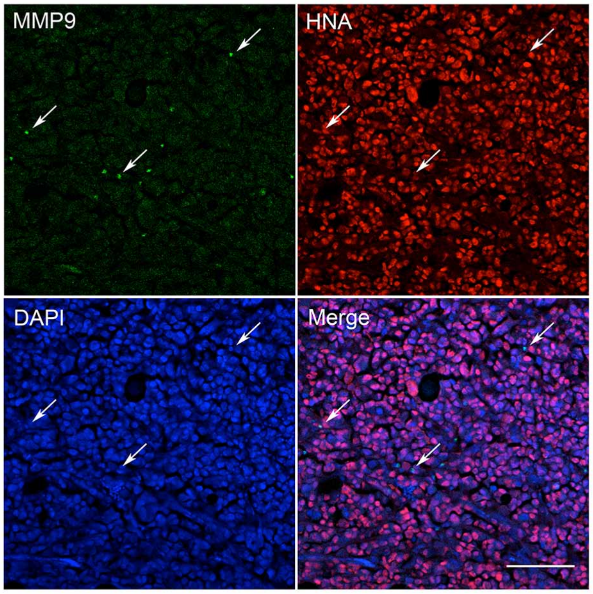

Supplementary Figure S7: MMP9 expression in tumors. Control tumor section showing MMP9 expression (tyramide signal amplified cyanine-3, pseudocolored green) in tumour cells overlaying with human nuclear antigen (HNA) expression (Alexa 647; red), counterstained with DAPI (blue). Arrows indicate MMP9 immunoreactivity adjacent to HNA-positive tumor cells. Scale bar, 100 µm.

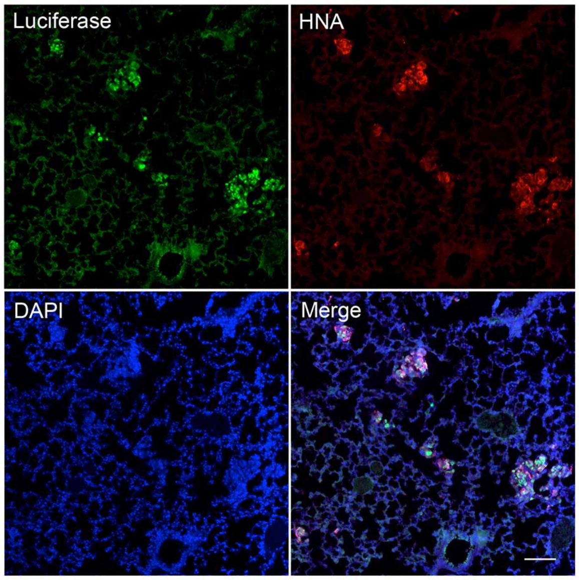

Supplementary Figure S8: Luciferase expression in metastases. Metastasis in lung section from a control tumour-bearing mouse showing luciferase expression (Alexa 488; green) in tumour cells overlaying with human nuclear antigen (HNA) expression (Alexa 568; red), counterstained with DAPI (blue). Scale bar, 100 µm.

Supplementary Table S1: Patient histoclinical characteristics and $Na_v1.5$ expression

Variable	$Na_v1.5$ expression		*P*
	Low (%)	High (%)	
Age			
≤ 50	8 (12.7)	17 (27.0)	0.99
> 50	13 (20.6)	25 (39.7)	
ER			
−	13 (20.0)	21 (32.3)	0.60
+	9 (13.8)	22 (33.8)	
Grade			
1	1 (1.5)	4 (6.2)	0.74
2	13 (20.0)	26 (40.0)	
3	8 (12.3)	13 (20.0)	
Menopausal status			
Premenopausal	7 (10.8)	21 (32.3)	0.29
Postmenopausal	15 (23.1)	22 (33.8)	
Node status			
−	12 (18.8)	15 (23.4)	0.19
+	10 (15.6)	27 (41.5)	
5-year BCa-specific survival			
Alive	19 (31.1)	39 (63.9)	0.27
Dead	2 (3.3)	1 (1.6)	

P values are from Fisher's exact tests except for grade, which is from χ^2 tests.

Supplementary Table S2: shRNA target sequences

Name	shRNA ID (Sigma/ Broad Institute)	Target sequence	Position on *SCN5A*	Match (%)
shControl	SHC002V	CAACAAGATGAAGAGCACCAA	-	0
shRNA1	TRCN0000043870	GCTGGACTTTAGTGTGATTAT	779	100
shRNA2	TRCN0000043871	GCCATCATCGTGTTCATCTTT	2733	100
shRNA3	TRCN0000043868	GCCGACAAGATGTTCACATAT	3915	100
shRNA4	TRCN0000043869	CCACTCAGTTTATTGAGTATT	5605	100

Supplementary Table S3: Primer sequences

Gene	Forward primer 5′-3′	Reverse primer 5′-3′	Reference
GAPDH	AAGGTGAAGGTCGGAGTCAAC	CCAGAGTTAAAAGCAGCCCTG	[1]
Adult $Na_v1.5$	CATCCTCACCAACTGCGTGT	ACATTGCCCAGGTCCACAAA	[2]
Neonatal $Na_v1.5$	CATCCTCACCAACTGCGTGT	AAAGTTCGAAGAGCCGACAA	[2]
β1	GTCGTCAAGAAGATCCACATTGAGGT	TTCGGCCACCTGGACGCCCGTGCAG	[3]

REFERENCES

1. Swift SL, Burns JE and Maitland NJ. Altered expression of neurotensin receptors is associated with the. Cancer Res. 2010; 70:347–356.
2. Brackenbury WJ, Chioni AM, Diss JK and Djamgoz MB. The neonatal splice variant of Nav1.5 potentiates *in vitro* metastatic behaviour of MDA-MB-231 human breast cancer cells. Breast Cancer Res Treat. 2007; 101:149–160.
3. Patino GA, Brackenbury WJ, Bao YY, Lopez-Santiago LF, O'Malley HA, Chen CL, Calhoun JD, Lafreniere RG, Cossette P, Rouleau GA and Isom LL. Voltage-Gated Na+ Channel beta 1B: A Secreted Cell Adhesion Molecule Involved in Human Epilepsy. J Neurosci. 2011; 31:14577–14591.
